# The Other Sibling: A Systematic Review of the Mental Health Effects on a Healthy Sibling of a Child With a Chronic Disease

**DOI:** 10.7759/cureus.29042

**Published:** 2022-09-11

**Authors:** Maria G Quintana Mariñez, Mohana Chakkera, Niriksha Ravi, Rajita Ramaraju, Aastha Vats, Athira R Nair, Atithi K Bandhu, Divya Koirala, Manoj R Pallapothu, Safeera Khan

**Affiliations:** 1 Pediatrics, California Institute of Behavioral Neurosciences & Psychology, Fairfield, USA; 2 Internal Medicine, California Institute of Behavioral Neurosciences & Psychology, Fairfield, USA; 3 Research, California Institute of Behavioral Neurosciences & Psychology, Fairfield, USA

**Keywords:** chronic conditions, psychosocial, chronic illness, mental health, siblings of chronic disease

## Abstract

The Center for Disease Control and Prevention (CDC) defines chronic diseases broadly as conditions that last over one year and require ongoing medical attention or limit activities of daily living or both. The diagnosis of a child with a chronic disease affects parents' mental health and functioning, included in this are the siblings of this child. The impact on a sibling of a child with chronic disease involves higher risks of anxiety, depression, feelings of worry about the brother or sister's future, and social problems.Three databases search were performed, and 16 articles were assessed in this systematic review that complies with inclusion and exclusion criteria.The siblings of those with chronic illnesses have higher reported emotional, behavioral, and social problems than those with healthy siblings. More research and studies with control groups and larger samples could contribute to a better understanding of the long-term effects of having a sibling with a chronic disease.

## Introduction and background

According to the Centers for Disease Control and Prevention (CDC), chronic diseases are defined broadly as conditions that last more than one year and require ongoing medical attention or limit activities of daily living or both [1]. The American Academy of Pediatrics states that the term chronic refers to a health condition that lasts anywhere from three months to a lifetime [2]. Consolini (2020) estimates that chronic health conditions affecting children range from 10 to 30% including asthma, cystic fibrosis, congenital heart disease, diabetes mellitus, attention-deficit/hyperactivity disorder, and depression. He also adds that chronic physical disabilities include meningomyelocele, hearing or visual impairments, cerebral palsy, and loss of limb function [3]. According to Anderson, in 2010, the most common chronic conditions present in children were asthma (30.4%) and other upper respiratory diseases that were not specified (36%) [4].

Children with chronic conditions face not only the effects of the conditions themselves but also issues related to mental health, anxiety and depression being the most common. Furthermore, the scope of disruption of daily life due to the disease affects school, friendships, extracurricular activities, social activities, and normal psychological development [5]. Cadman in 1987 reported the results of a large epidemiologic survey, and those with chronic diseases and disabilities had a threefold risk for psychiatric disorders and increased risk for social problems [6]. More recently, in 2022, children with chronic health conditions were approximately twice as likely at 10 and 13 to present with a mental health disorder as the control group [7].

The parents of children with chronic illnesses are also deeply affected, physically they are tired, with little energy, and mentally frustrated, anxious, angry, helpless, and hopeless. Cognitive problems are present such as remembering tasks, worrying not only for their child and the illness itself but about their child's future, and the effect of the condition on other members of the family. Social isolation was another complaint [8]. Parents complained about feeling exhausted caused by the necessity of constantly having to care for their children. They believed their entire lives were affected by their child’s illness [9].

Parents have an important role, healthy siblings of those with chronic diseases are key. Göbel (2016) discussed Ihle's (2000) findings on internalizing problems for children in general, which include anxiety and depression, social withdrawal, and somatic complaints [10,11]. Externalizing behavior problems are characterized by delinquent, oppositional, and aggressive conduct. Long-term consequences are problems within social, school, and later professional environments [10]. Caring responsibilities were common among adolescent siblings of young people with chronic illness and there was an important prevalence of clinically relevant distress, anxiety, depression, anger, and need for help among the siblings [12]. Children with highly intrusive and/or life-threatening chronic health conditions appear especially at risk for psychological problems and especially vulnerable to internalizing problems [13,14].

Although research about healthy siblings has increased in the last years, most studies are focused on whole family experiences, parents or sick child experiences, and/or their well-being. There is still a gap in knowledge about the mental health of siblings without chronic disease (healthy sibling). For this reason, this paper is focused only on this population with the following research question: what are the effects on the mental health of healthy siblings who have a sibling with a chronic disease?

## Review

Methods

For this review, the 2020 Preferred Reporting Items for Systematic Reviews and Meta-Analyses (PRISMA) guidelines were used [15]. Eligibility criteria were chosen for this review such as: including papers from the past ten years, papers written in English, including the pediatric population (up to 21 years of age), and articles relevant to the topic. Exclusion criteria were articles including adult population, papers written in other languages other than English, unpublished papers, and grey literature.

Search Strategy

Four databases were utilized: PubMed, Cochrane Library, Popline, and Google Scholar. The search was performed between June 4 and June 10, 2022. Keywords were created separating the main subjects into concepts, resulting in: “siblings of chronic disease AND mental health” OR “siblings AND chronic illness AND psychosocial” OR “siblings AND chronic conditions AND mental health”. A medical subject headings term (MeSH) strategy for PubMed search was also applied, shown in Appendix 1. Search results are shown below in Table 1 with results before and after applying filters.

**Table 1:** All database research results. Last search June 10, 2022.

	PubMed	Cochrane	Popline	Total
Initial search results	115	45	176	336
Final search results applying filters (2012-2022, population up to 21 years old).	58	33	93	184

Study Selection and Data Collection

All results from the search were transferred to EndNote online citation manager (Clarivate). Duplicates were removed manually by reading titles and abstracts. The articles that were relevant to our topic were selected, for those in which only abstracts were available an email requesting an author’s copy was sent. Articles that in full were entirely read and during this process, inclusion and exclusion criteria were thoroughly checked. 

Bias Risk and Study Quality Assessment

The risk of bias was assessed by two independent researchers who evaluated each study independently and were later discussed. All articles underwent quality assessment using the appropriate tools pertinent to each type of study. The New Castle Ottawa tool was used for observational studies (Table 2) [16], the Appraisal tool for cross-sectional studies (AXIS) (Table 3) [17], and the AMSTAR checklist for systematic reviews (Table 4) [18].

**Table 2:** Quality assessment and risk of bias for observational studies using The New Castle Ottawa tool [16].

Study	Selection	Comparability	Outcome	Overall (max. 9)
Shojaee et al. [19]	*****	0	*	7, Good
Caliendo et al. [20]	****	0	**	6, Medium
Pourbagheri et al. [21]	****	*	*	7, Good
Fullerton et al. [22]	****	0	**	6, Medium
Haukeland et al. [23]	*****	0	**	6, Medium
Dinleyici et al. [24]	****	*	***	8, Good
Emerson et al. [25]	*****	*	***	9, Good

**Table 3:** Quality assessment and risk of bias for cross-sectional studies using AXIS [17].

Study	Introduction	Methods	Results	Discussion	Others	Score	Quality
Kelada et al. [12]	1/1	9/10	4/5	2/2	2/2	19/20	Good
Chan et al. [26]	1/1	8/10	3/5	2/2	1/2	15/20	Good
Nasr et al. [27]	1/1	8/10	3/5	2/2	1/2	16/20	Good
Velleman et al. [28]	1/1	8/10	3/5	2/2	2/2	17/20	Good
Fleary et al. [29]	1 /1	9/10	3/5	2/2	1/2	16/20	Good
Petalas et al. [30]	1/1	7/10	4/5	2/2	2/2	16/20	Good
Fredriksen et al. [31]	1/1	8/10	4/5	2/2	2/2	18/20	Good

**Table 4:** Quality assessments and risk of bias of meta-analyses and systematic review studies using AMSTAR [18]. Legend: Y= YES PY= Partial Yes NMA= No meta-analysis was conducted.

Questions																	
Study	1	2	3	4	5	6	7	8	9	10	11	12	13	14	15	16	Score
Shivers et al. [32]	Y	Y	Y	Y	Y	Y	PY	PY	Y	Y	Y	Y	Y	Y	Y	Y	High
Piotrowski et al. [33]	Y	Y	Y	PY	Y	Y	Y	Y	Y	Y	NMA	NMA	Y	Y	NMA	Y	Moderate

Results

**Figure 1:**
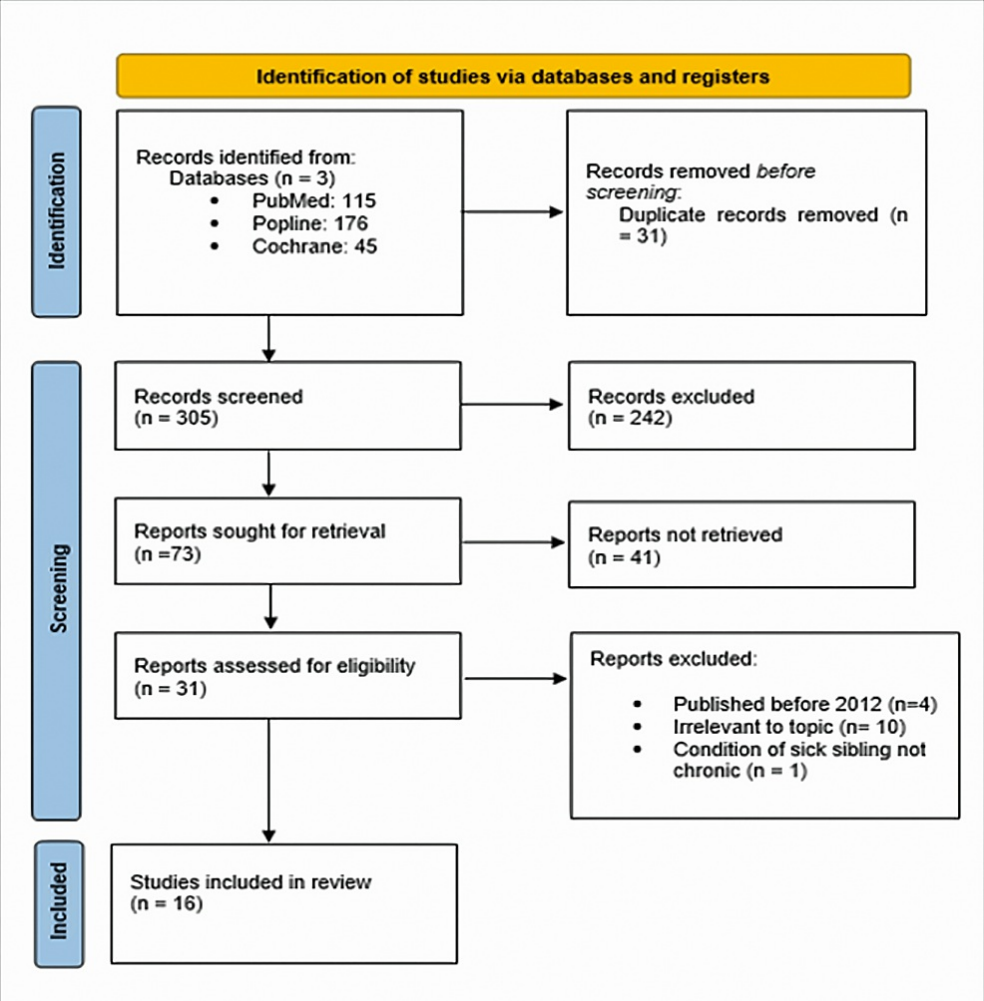
Identification of studies via databases and registers.

A total of 336 articles were identified from all databases used during the search: 115 from PubMed, 176 from Popline, and 45 from Cochrane Library. Thirty-one duplicates were manually removed. After reading the title and abstract 242 were found not relevant to the topic and were deleted. A total of 73 articles were sought to retrieve of which 43 were not retrieved and 31 were full texts from a website or directly through the author. From those found during the retrieval process 3 were removed because were published before 2012, 10 were irrelevant and 1 was not a chronic condition. The final count was 17 articles chosen for quality assessment: 14 observational studies, 1 systematic review, and 2 meta-analyses. A PRISMA [15] flow diagram is shown in Figure 1, and baseline characteristics of selected studies are described in Table 5. Both are found below.

**Table 5:** Baseline characteristics of selected studies.

Reference/Article	Region	Type of study	Sample size/Age range	Conclusion
Shojaee et al. (2019) [19]	Iran	Observational	91 siblings. 38 siblings of children with sensory impairment and 58 without. 10-18 years of age.	Greater emotional and peer relationship problems than control.
Caliendo et al. (2020) [20]	Italy	Observational	159 siblings (53 siblings of children with Autism, 53 siblings of children with Down syndrome, and 53 siblings of healthy children). 3-9 years of age.	More emotional difficulties in siblings who have autism and Down syndrome than in those with healthy siblings. Especially in males.
Pourbagheri et al. (2018) [21]	Iran	Observational	174 siblings (58 siblings of children with autism, 58 siblings of children with Down syndrome, and 558 siblings of healthy children). 3-9 years of age.	Higher emotional and behavioral distress in siblings of children with diseases, more in siblings of autistic children. No male and female difference. More problems in ages 3-7 years in siblings of children with Down syndrome.
Fullerton et al. (2017) [22]	United Kingdom	Observational	39 siblings of children with life-limiting conditions and 32 siblings of children with autism. Age 10-16 years.	Higher psychological problems in siblings of children with life-limiting conditions are similar to those with autistic siblings. Lower quality of life.
Piotrowski et al. (2022) [33]	Canada	Systematic review.	8 studies.	Siblings of children with chronic kidney disease are negatively affected in several ways: psychosocial wellness and family. Small size studies were not significant. Larger quantitative studies showed more anxiety symptoms but not depression or traumatic stress symptoms.
Dinleyici et al. (2020) [24]	Turkey	Observational	191 siblings of children with chronic disease and 100 siblings of healthy children. Age 2-18 years.	Children with chronically diseased siblings have more physical and psychological problems. More self-reported decreases in quality of life than those reported by parents.
Haukeland et al. (2021) [23]	Norway	Observational	56 siblings of children with rare disorders and 44 controls. Age 8-16 years.	Lower self-reported mental health compared to controls. Poorer communication with parents and social support. No difference in the anxious relationship with the mother and parent-reported mental health problems of a healthy sibling.
Chan et al. (2016) [26]	China	Cross-Sectional	116 siblings of children with an autism spectrum disorder. Age 6-18 years.	One quarter is at risk of mental health problems. Parents reported emotional and behavioral problems with no difference. Weak peer relationships and prosocial behavior.
Emerson et al. (2014) [25]	Australia	Observational	8571 siblings of children with chronic disease or disability. 7636 living with a sick sibling at 4/5 years of age and 6/7 years of age (waves). 1232 living with a sibling with long-term health condition at both waves and 268 living with a sibling with disability at both waves.	Higher risk of low overall well-being in children at 4/5 years of age more than 6/7 years of age when economic and social difficulties are considered. No significant deterioration from one wave to the other.
Kelada et al. (2021) [12]	Australia	Cross-Sectional	45 siblings Age >12 years	Lower physical and psychosocial well-being was reported. Higher coping skills, lower anxiety, and need for help when siblings had caring responsibilities.
Nasr et al. (2018) [27]	Iran	Cross-Sectional	60 siblings: 30 siblings of children with autism spectrum disorder and 30 children with siblings with chronic disease. Age 4-11 years.	No statistical difference among groups in anxiety, depression, rule-breaking behavior, and attention problems. Aggressive behavior statistically different.
Velleman et al. (2016) [28]	United Kingdom	Cross-Sectional	55 siblings of children with chronic fatigue syndrome or myalgic encephalomyelitis. Age 12-17 years	Higher anxiety scores but not depression. Females with higher separation and social anxiety. Negative impact on family roles and communication.
Petalas et al. (2012) [30]	United Kingdom	Cross-Sectional	12 healthy siblings of children with an autism spectrum disorder. Age 14-17 years.	Adolescents often worry about the future of their siblings. Anxious feelings about aggressive behavior of sibling, embarrassment. Sense of introspection and empathy are not found in younger siblings.
Fleary et al. (2013) [29]	United States	Cross-Sectional	40 siblings of children with chronic disease. Age 18-21 years.	>60% of siblings with clinically normal or low psychosocial problems. Some problems such as acting out, hostility, and social withdrawal are clinically significant but the total score is similar to the control. Males have more behavior problems and females have more trusting issues.
Fredriksen et al. (2021) [31]	Norway	Cross-Sectional	107 siblings of children with chronic disease and 199 parents of children with chronic disease. Age >11 years.	Mental health problems are under clinical cutoff but 1/6 siblings and 1/3 of parents reported being at least borderline. Fathers reported lower mental health problems than mothers. Parent and child quality of communication is an important predictor of mental health. No difference in father and mothers’ reported siblings’ mental health. No gender difference.
Shivers et al. (2018) [32]	United States	Meta-analysis	69 independent samples.	Siblings of children with autism are more likely to have negative beliefs (mainly about themselves). Worse sibling relationship. More anxiety, depression and attention deficit and hyperactivity disorder symptoms.

Discussion

Shojaee et al. compared the emotional and behavioral difficulties in healthy siblings of children with and without sensory impairment. The sample was composed of 91 children between 10 and 18 years old, 38 were siblings of children with sensory impairments and 53 were controls. Authors found more emotional difficulties in those with a sibling with sensory impairment than those without it. Additionally, they also had trouble with peer relations skills. For all subscales of emotional problems, no difference was found between males and females for those siblings of sick children [19].

In contrast, Caliendo et al. chose a bigger sample (159 healthy siblings), 3-9 years old (mean age 5.49), and decided to divide it into three groups: 1. Healthy siblings of children with Down syndrome (N=53), 2. Healthy siblings of children with autism (N=53) and 3. Healthy siblings of children with typical development (N=53) [20]. As an important point, both studies used the same method to measure their outcomes, which was the strength and difficulties questionnaire (SDQ) assessing emotionality, behavioral problems, hyperactivity and inattention, peer relationship, and pro-social behavior. In this case, results were concordant with the presumption that healthy siblings are at risk of developing emotional problems. Males were more likely to have emotional issues when compared to females however, there was no age disparity [20]. Contrary to Shojaee et al. in which no major findings between males and females were reported [19].

Pourbagheri et al. divided the sample into three groups: autism, Down syndrome, and the control group, and separated them by age into a 3-7-year-old group and a 7-9-year-old group [21]. The selection of groups with diseases clearly defined gives a better understanding of the relationship between those specific conditions and the emotional health of healthy siblings [20,21]. The SDQ score for the Down syndrome and autism group did not show an important clinical difference, but a significant statistical difference between healthy siblings of children with the above conditions compared to siblings of healthy children was found [20]. On the other hand, Pourbagheri et al. reported a higher overall score in the autistic group (63.8%) compared to the other two groups, but these groups were separated by age and compared to each other, the 3-7 years old in the Down syndrome group showed more emotional behavioral problems than the 7-9 years old group, while in the autism group the 3-7 years old had more difficulties only in the peer subscale compared to the 7-9-year-old group [21]. 

Moreover, in the hyperactivity and inattention subscale, no difference was found [19] but Caliendo et al. and Pourbagheri et al. noticed more difficulties such as problems concentrating, impulsivity, restlessness, or the inability to stand still in the autism and Down syndrome group compared to control [20,21]. This difference was greater in the autism group when compared to Down syndrome and the control group but not when compared by age. Also, peer relationship problems were greater than the control group for both studies, although healthy siblings of autistic children between 3-7 years old showcased greater problems with peers than the 7-9 years old group [21].

Siblings of autistic children have more prosocial behavior skills than siblings of children with Down syndrome but also more adaptation problems [21]. On the contrary, in Hong Kong, in an observational study of 116 healthy siblings of children with autism, the SDQ score was higher compared to population norms but no difference in behavioral and emotional problems was reported, however, cases (siblings of autistic children) had more problems in the prosocial subscale [26]. 

In the conduct or behavioral subscale, Shojaee et al. did not find a difference while Caliendo et al. found for the control group 7.5% of clinical discomfort versus 26.4% for the Down syndrome and autistic group [19,20]. Closely, Nasr et al. (2018) compared 30 4-11 years-old healthy siblings of children with autism in Iran with 30 siblings of children with chronic physical illnesses and concluded that problems such as anxiety, depression, social and attention problems as well as rule-breaking behavior were not different between groups, yet aggressive behavior was statistically significant [27].

A meta-analysis done by Shivers et al. about siblings of children with autism also noticed more problems such as anxiety and depression, attention deficit and hyperactivity disorder (ADHD), and externalizing problems, for example, aggressive behavior, property damage, and theft [32]. Similarly, Fullerton et al. in 2017, sampled 39 siblings in the range of 3-16 years old, mean age of 8.23, and compared healthy siblings of children with conditions that limit life with population norms against 32 siblings of children with autism. In concordance with the aim of this review, their results support the hypothesis that healthy siblings have a great level of emotional symptoms, conduct problems, hyperactivity, as well as behavioral difficulties. As an important note, the author accounts for the corresponding similarity of chronic conditions and life-limiting diseases yet accounts for the distinction that a life-limiting condition is not always chronic [22].

Siblings of children with rare disorders might benefit from timely treatment and intervention [23,28]. In 2021, of 100 siblings, 56 were chosen as cases and 44 controls, with ages ranging from 8 to 16 years old, mean age of 11.5 years old were compared, in this case, the diseases selected were 21 rare disorders, the majority were chromosomal disorders such as Duchenne muscular dystrophy, Prader-Willi syndrome, Noonan syndrome, and Fragile X syndrome, among others [23]. Results were agreeable with previous studies in terms of rare diseases and mental health such that Velleman (2016) in the United Kingdom with a sample of 32 siblings (age 12-17) of children with chronic fatigue syndrome or myalgic encephalomyelitis (CFS/ME) concluded that siblings are situated in the 90th percentile for anxiety including generalized, separation anxiety, social anxiety, panic, and obsessive-compulsive behaviors among others but not depression [28]. Results are comparable with Piotrowski et al. (2022) for children with siblings with chronic kidney disease, siblings displayed more anxiety, in studies with larger samples compared to small size samples. Depression or traumatic stress symptoms suggested by previous studies included in their review were not found of high importance, but the authors recognize that the self-reporting method could be the cause of variability in the results. [33].

In Turkey, a large observational study with 191 siblings and 100 controls, evaluated the quality of life of healthy siblings of those with chronic disease and as previous authors did, specific diseases were investigated: cerebral palsy, hematologic/oncologic disease, asthma, type 1 diabetes, celiac disease, and epilepsy. In this case, part of the investigation included their psychosocial health comprehended by emotional, social, and school functioning. Healthy siblings of children of all above mentioned diseased had lower psychosocial scores when compared individually to the control group, especially for those with siblings with cerebral palsy. Nevertheless, when combined, there was no significant difference among cases and controls, suggesting that some diseases have more effect than others and they should be compared separately [24]. Proper adjustment for other factors such as family composition, parental mental health, socioeconomic status, and environmental hardships are often necessary for comparison [24,25].

Fleary et al. focused their research on adolescence and the impact of growing up with a chronically ill sibling in an observational study comprised of 40 participants 18-21 years of age attending university. In this matter, the authors concluded that this group of age might experience clinically significant problems such as acting out, psychotic features, social withdrawal, and hostile behavior. Communication problems often arise particularly with parents, possibly affecting social interaction and functioning in adulthood [29]. Adolescence and early childhood years seem to be a critical period of development and pose higher risks of emotional and behavioral problems [21,25]. Adolescents between 14-17 years of age are often worried about the future of their respective siblings [30].

In 2021 Fredriksen et al. studied the predictors of mental health in siblings with 107 siblings, a mean age of 11.5 years to effectively assess which siblings could be at risk of mental health problems. How the sibling in question adjusts to their brother's or sister's disease had important significance. A significant factor was the parent-child relationship more than parent-child communication, the authors recognized this could represent a potential weakness related to their data collection design. Fathers' mental health did not show as significant importance as mothers’ mental health, explaining this could be related to lower reporting thresholds from fathers, pointing to the importance of including them as an important part of future research [31]. 

Limitations and strengths 

In brief, the limitations of our study include the data gathering. We could only identify 16 articles that met our inclusion criteria. Also, the articles and their aims were too broad, and covered areas that were not relevant to our research question for which data extraction was complex. 

The strengths of this study are comprised of articles with different sample sizes including large sizes and meta-analyses. Additionally, the range of diseases included was satisfactory to cover the concepts of chronic diseases, yet chronic rare diseases were also mentioned. 

## Conclusions

To sum up, in line with the research question, the study concludes that healthy siblings of children with chronic diseases show more susceptibility to emotional and behavioral difficulties. Importantly, the sphere of problems is broad and includes trouble with emotions, peer relationships, conduct, hyperactivity and inattention, and prosocial behavior. Results among studies in this review are mixed in which type of problem was clinically and statistically relevant and we suspect this variability is influenced by environmental factors not addressed in this article, such as family composition, familial mental health, and socioeconomic status, among others. Also, most psychological problems were clinically classified as borderline. Additionally, age seems to be a critical factor and young children appear to be more affected than older children. Only two studies focused their attention on late adolescence which is also a crucial developmental period of life. We encourage further research on this topic especially in late adolescence and young adulthood with an emphasis on predictors and identification of risk factors in large populations, to improve the detection of children at high risk of developing clinically significant mental health problems such as mood disorders and to develop better strategies and guidelines for timely support, intervention, and monitoring of healthy siblings.

## Appendices

Appendix 1: MeSH search strategy

((( "Siblings/epidemiology"[Mesh] OR "Siblings/ethnology"[Mesh] OR "Siblings/psychology"[Mesh] OR "Siblings/statistics and numerical data"[Mesh] )) AND (( "Chronic Disease/analysis"[Mesh] OR "Chronic Disease/chemistry"[Mesh] OR "Chronic Disease/classification"[Mesh] OR "Chronic Disease/complications"[Mesh] OR "Chronic Disease/congenital"[Mesh] OR "Chronic Disease/diagnosis"[Mesh] OR "Chronic Disease/economics"[Mesh] OR "Chronic Disease/epidemiology"[Mesh] OR "Chronic Disease/ethnology"[Mesh] OR "Chronic Disease/etiology"[Mesh] OR "Chronic Disease/genetics"[Mesh] OR "Chronic Disease/history"[Mesh] OR "Chronic Disease/immunology"[Mesh] OR "Chronic Disease/legislation and jurisprudence"[Mesh] OR "Chronic Disease/methods"[Mesh] OR "Chronic Disease/mortality"[Mesh] OR "Chronic Disease/organization and administration"[Mesh] OR "Chronic Disease/parasitology"[Mesh] OR "Chronic Disease/physiology"[Mesh] OR "Chronic Disease/physiopathology"[Mesh] OR "Chronic Disease/prevention and control"[Mesh] OR "Chronic Disease/psychology"[Mesh] OR "Chronic Disease/secondary"[Mesh] OR "Chronic Disease/statistics and numerical data"[Mesh] OR "Chronic Disease/trends"[Mesh] ))) AND (( "Mental Health/classification"[Mesh] OR "Mental Health/diagnosis"[Mesh] OR "Mental Health/drug effects"[Mesh] OR "Mental Health/economics"[Mesh] OR "Mental Health/education"[Mesh] OR "Mental Health/epidemiology"[Mesh] OR "Mental Health/ethnology"[Mesh] OR "Mental Health/history"[Mesh] OR "Mental Health/legislation and jurisprudence"[Mesh] OR "Mental Health/methods"[Mesh] OR "Mental Health/nursing"[Mesh] OR "Mental Health/organization and administration"[Mesh] OR "Mental Health/prevention and control"[Mesh] OR "Mental Health/standards"[Mesh] OR "Mental Health/statistics and numerical data"[Mesh] OR "Mental Health/therapy"[Mesh] OR "Mental Health/trends"[Mesh] ))
